# An insight into the sialotranscriptome of the West Nile mosquito vector, *Culex tarsalis*

**DOI:** 10.1186/1471-2164-11-51

**Published:** 2010-01-20

**Authors:** Eric Calvo, Irma Sanchez-Vargas, Amanda J Favreau, Kent D Barbian, Van M Pham, Kenneth E Olson, José MC Ribeiro

**Affiliations:** 1 Section of Vector Biology, Laboratory of Malaria and Vector Research, National Institute of Allergy and Infectious Diseases, National Institutes of Health, Rockville MD 20852, USA; 2 Department of Microbiology, Immunology, & Pathology, Colorado State University, Fort Collins, CO 80523, USA; 3 Genomics Unit, Research Technologies Section, Rocky Mountain Laboratories, Hamilton, MT 59840, USA

## Abstract

**Background:**

Saliva of adult female mosquitoes help sugar and blood feeding by providing enzymes and polypeptides that help sugar digestion, control microbial growth and counteract their vertebrate host hemostasis and inflammation. Mosquito saliva also potentiates the transmission of vector borne pathogens, including arboviruses. *Culex tarsalis *is a bird feeding mosquito vector of West Nile Virus closely related to *C. quinquefasciatus*, a mosquito relatively recently adapted to feed on humans, and the only mosquito of the genus *Culex *to have its sialotranscriptome so far described.

**Results:**

A total of 1,753 clones randomly selected from an adult female *C. tarsalis *salivary glands (SG) cDNA library were sequenced and used to assemble a database that yielded 809 clusters of related sequences, 675 of which were singletons. Primer extension experiments were performed in selected clones to further extend sequence coverage, allowing for the identification of 283 protein sequences, 80 of which code for putative secreted proteins.

**Conclusion:**

Comparison of the *C. tarsalis *sialotranscriptome with that of *C. quinquefasciatus *reveals accelerated evolution of salivary proteins as compared to housekeeping proteins. The average amino acid identity among salivary proteins is 70.1%, while that for housekeeping proteins is 91.2% (P < 0.05), and the codon volatility of secreted proteins is significantly higher than those of housekeeping proteins. Several protein families previously found exclusive of mosquitoes, including only in the *Aedes *genus have been identified in *C. tarsalis*. Interestingly, a protein family so far unique to *C. quinquefasciatus*, with 30 genes, is also found in *C. tarsalis*, indicating it was not a specific *C. quinquefasciatus *acquisition in its evolution to optimize mammal blood feeding.

## Background

Most adult female mosquitoes are hematophagous, in addition to taking sugar meals. Saliva helps blood feeding by interfering with host reactions that could disrupt the blood flow, assists sugar meals with glycosidases, and contain antimicrobials that may control microbial growth in their meals [1]. With the advent of tissue transcriptomics, we can postulate that these functions are mediated by large numbers (70-100) of polypeptides, many of which are expressed solely in the adult female salivary glands [2]. Unique protein families have been found in *Anopheles*, *Aedes *or *Culex *mosquitoes, as well as a group of common proteins or enzymes [3-5]. Functional characterization of these proteins uncovers scavengers of biogenic amines [6,7] or leukotrienes [8], inhibitors of blood clotting [9-11], bradykinin formation [12,13], platelet aggregation [14,15] and vasodilators [16,17]. Other molecularly uncharacterized activities include inhibitors of mast cell TNF production [18] and inhibition of T cell activation [19,20]. It is apparent that the complexity of the salivary components affecting host hemostasis and inflammation mirrors the complexity of host hemostasis and inflammation itself, which must be disarmed for successful blood feeding. Indeed mosquitoes lacking salivation by salivary duct ablation feed less and take more dangerous time of exposure to their hosts [21,22].

Perhaps due to the potent pharmacological activities of saliva, or the immune reactions to it, mosquito saliva also plays a role in pathogen transmission, including in arboviral transmission [23-25]. Accordingly, determination of the salivary composition of vector mosquitoes not only discovers new potentially pharmacologically active molecules, but also can also help generating vaccine targets for disruption of arboviral transmission. These proteins may also be of epidemiological significance as selective human or animal markers of vector exposure [26-29].

We have previously described the sialotranscriptome of *C. quinquefasciatus *[5], where several unique protein families were discovered, many of which are products of gene duplications. Indications of horizontal gene transfer from bacteria to mosquitoes were also pointed out as participating in the generation of mosquito sialomes [3,4]. To this date, no other sialotranscriptome from a member of the Culex genus has been described. We currently portray the sialotranscriptome of *Culex tarsalis*, a North American bird feeding mosquito [30], and, like *C. pipiens*, a good vector of West Nile virus [31].

## Results and Discussion

### Characteristics of the assembled salivary EST set

A total of 1,753 cDNA clones were used to assemble a database (Additional file 1, Table S1) that yielded 809 clusters of related sequences, 675 of which contained only one expressed sequence tag (EST). The 809 clusters were compared, using the programs BlastX, BlastN, or RPSBLAST [32], to the nonredundant protein database of the National Center of Biological Information (NCBI), to a gene ontology database [33] to the conserved domains database of the NCBI [34], and to a custom prepared subset of the NCBI nucleotide database containing either mitochondrial or rRNA sequences.

**Figure 1:**
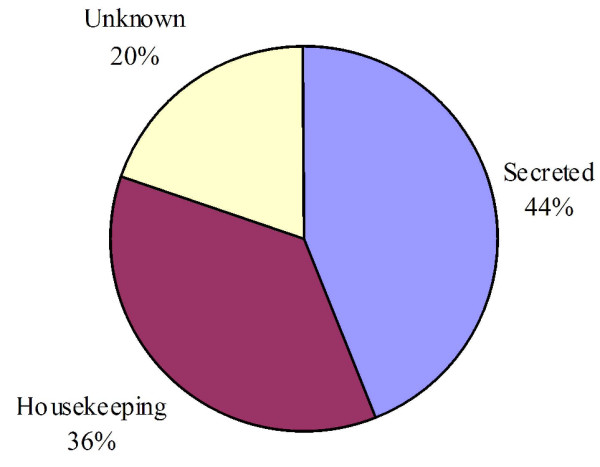
**Distribution of the transcripts from the salivary gland cDNA library of *Culex tarsalis *according to functional class**.

Three categories of expressed genes derived from the manual annotation of the contigs (Fig 1). The putatively secreted (S) category contained 44% of the sequences, the housekeeping (H) category had 36.3% and 19.7% of the ESTs could not be classified and belong to the Unknown (U) class. The transcripts of the unknown class could represent novel proteins or derive from the less conserved 3' or 5' untranslated regions of genes, as was indicated for the sialotranscriptome of *An. gambiae *[3]. This distribution is typical of previous mosquito sialotranscriptomes [4,5,35-37].

### Housekeeping (H) genes

The 637 ESTs attributed to H genes expressed in the salivary glands of *C. tarsalis *were further characterized into 19 subgroups according to function (Table 1 and Additional file 1, Table S1). Transcripts associated with the protein synthesis machinery represented 44% of all transcripts associated with a housekeeping function, an expected result for the secretory nature of the organ. Nineteen percent of the transcripts were classified as either "Unknown conserved" or "Conserved secreted proteins". These represent highly conserved proteins of unknown function, presumably associated with cellular function, but still uncharacterized. These sets may help functional identification of the "Conserved hypothetical" proteins as previously reviewed in [38]. Transporters accounted for 7% and included several members of the vacuolar ATPase machinery, important for mosquito salivary secretion [39]. Energy metabolism and protein modification related enzymes accounted for nearly 5% each of the transcripts of the H class.

**Table 1:** Classification and relative accumulation of *Culex tarsalis *salivary glands transcripts that are associated with housekeeping function

Class	Number of transcripts	Percent of housekeeping group
Protein synthesis machinery	279	43.8
Unknown conserved	120	18.8
Transporters and storage	44	6.9
Energy metabolism	30	4.7
Protein modification machinery	29	4.6
Amino acid metabolism	21	3.3
Signal transduction	17	2.7
Carbohydrate metabolism	15	2.4
Protein export machinery	15	2.4
Cytoskeletal	11	1.7
Transcription machinery	11	1.7
Lipid metabolism	10	1.6
Proteasome machinery	10	1.6
Extracellular matrix	7	1.1
Nuclear regulation	6	0.9
Immunity	4	0.6
Intermediate/secondary metabolism	4	0.6
Nucleic acid metabolism	2	0.3
Transcription factors	2	0.3
		
**Total**	637	

### Possibly secreted (S) class of expressed genes

A total of 771 ESTs represent putative *C. tarsalis *salivary components (Tables 2 and Additional file 1, Table S1). These include ubiquitous known gene families, e.g, families seen in other organs and organisms, as well as protein families unique to hematophagous Diptera, or to Culicines, or to *Culex *genus alone. Table 2 also indicates our degree of knowledge, or ignorance, regarding these protein families, 15 of which we have no hint for their function, more than half of the 24 classes of proteins represented in Table 2. Many of these putatively secreted protein families of unknown function are multigenic, such as the CWRP family found previously only in *C. quinquefasciatus *[5], which contributes to 58% of the ESTs associated with the S class in *C. tarsalis*. The D7/OBP-like families contribute to 9% of all transcripts associated with secreted products, which is less than previously seen in *Anopheles *or *Aedes *sialotranscriptomes, probably due to the dilution effect following abundant expression of the CWRP family.

**Table 2:** Classification and relative accumulation of *Culex tarsalis *salivary glands transcripts that are associated with secreted products

Class	Number of transcripts	Percent of secreted group	Function known or presumed? *
Subclass			
**Ubiquitous protein families**			
Secreted Enzymes			
Amylase/maltase/chitinase	84	10.9	Y
Serine proteases	16	2.1	Y/N
Adenosine deaminase	4	0.5	Y
Phosphatase	3	0.4	N
DNAse	1	0.1	Y
Salivary immunity related products	7	0.9	Y
Antigen 5 family	5	0.6	N
Mucins	3	0.4	Y
Serpin	1	0.1	Y
**Unique hematophagous Diptera proteins**			
D7/OBP family	66	8.6	Y
30 kDa antigen/Aegyptin	20	2.6	Y
6.3 kDa family	11	1.4	N
**Uniquely Culicine families**			
30.5 kDa family	40	5.2	N
23.4 kDa family	2	0.3	N
41.9 kDa family	2	0.3	N
62 kDa family	1	0.1	N
Fragment of culicine salivary protein	1	0.1	N
**Uniquely Culex families**			
16.7 kDa family	443	57.5	N
GQP repeat family	32	4.2	N
9.7 kDa family	16	2.1	N
4.2 kDa family	6	0.8	N
HHI repeat family	4	0.5	N
Cys rich family	2	0.3	N
7.8 kDa family	1	0.1	N

* Y for Yes and N for No - Yes is probably an overestimation as proteins can be multi functional

### The salivary secretome of *Culex tarsalis*

From the sequenced cDNAs, a total of 283 novel *C. tarsalis *protein sequences were derived, 80 of which code for putative secreted products (Additional file 2, **Table S2**). Because cDNA sequences coding for many of these proteins were found as singletons, this secretome is to be considered preliminary and incomplete, but many parallels with previous mosquito sialotranscriptomes can be drawn, as follows:

### Proteins with presumed or experimentally validated function

#### The D7/Odorant binding protein-like family

The D7 proteins constitute a unique multi gene family found expressed in the salivary glands of blood sucking Diptera [40], belonging to the superfamily of Odorant Binding Proteins (OBP) [41]. Long and short versions exist, the long versions containing two and the short versions containing one OBP domain. Three genes codes for long D7 proteins and 5 genes code for short D7 proteins in *An. gambiae *[3], while in *Ae. aegypti *2 genes code for long and 3 genes code for short D7 proteins [4]. Short versions of anopheline D7 mosquitoes were shown to bind biogenic amines such as serotonin and histamine [6], thus counteracting hemostasis and inflammation. More recently, the amino terminal OBP domain of a D7 long form of *Ae. aegypti *was shown to bind peptidic leukotrienes with high affinity. The crystal structures of a short D7 protein from *An. gambiae *and a long D7 protein from *Ae. aegypti *revealed that the D7 OBP domains have 7 alpha helices, 2 more than the canonical OBP family [7]. In addition to these inflammatory agonist binding functions, a short D7 protein from *An. stephensi*, named hamadarin, was shown to inhibit bradykinin formation by inhibiting the FXII/Kallikrein pathway [12].

**Figure 2:**
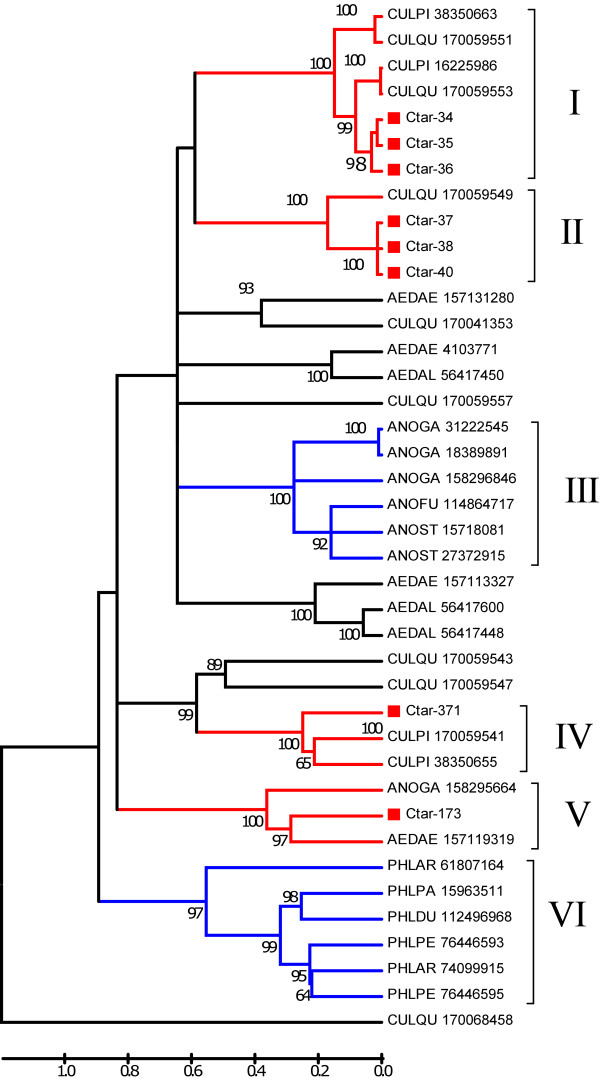
**Phylogram of the D7 protein family of mosquitoes**. The numbers on the tree nodes represent the percent bootstrap support in 10,000 trials (only values above 50% are shown). The bar at the bottom indicates amino acid divergence. The *Culex tarsalis *sequences are named Ctar-XXX where the XXX represents the cluster number that originated it. The remaining sequences are named in a five letter followed by number format where the 3 first letters represent the 3 first letters of the genus, followed by the first 2 letters of the species binomial name. The number represents the NCBI gi| access. For more details, see text.

Eight members of the D7 protein family were found in the *C. tarsalis *sialotranscriptome, in addition to two canonical OBP proteins. Alignment of these proteins with their close blast matches found in the NR protein database, and construction of a bootstrapped phylogram (Fig 2) shows several clades with strong bootstrap support, and allows the following inferences: The eight *C. tarsalis *proteins are the probable results of 4 genes. Ctar-34, Ctar-35 and Ctar-36 are possible alleles of a single gene related to other long *C. quinquefasciatus *D7 proteins shown in Clade I, while Ctar-37, Ctar-38 and Ctar-40 are probably alleles of a second *C. tarsalis *gene coding for yet another long D7 protein, and are associated within Clade II. Ctar-173, results from a gene coding for a third long D7 protein grouping in Clade V with other mosquito long D7 proteins, including, *Anopheles *and Aedes *proteins*. Ctar-371 is the only *C. tarsalis *sequence for a short D7 protein, grouping in clade IV with other *Culex pipiens *D7 proteins. Notice also Clades III and Clade VI, which exclusively contain long D7 Anopheline or Phlebotomine proteins, respectively.

Ctar-34, Ctar-35, Ctar-37 and Ctar-38 possess the signature [ED]- [EQ]-x(7)-C-x(12,17)-W-x(2)-W-x(7,9)- [TS]-x-C- [YF]-x- [KR]-C-x(8,22)-Q-x(22,32)-C-x(2)- [VLI] found in lipid binding D7 domain of *Ae. aegypti *[8]. The serotonin binding motif found on short anopheline D7 proteins as well as the carboxydomain of the long D7 protein of *A. aegypti *[6,7] was not found on any *C. tarsalis *D7 protein (nor in any D7 protein of *C. quinquefasciatus *- results not shown), indicating this motif may have evolved beyond recognition, or that other proteins in *Culex *may undertake this task (see below under CWRP heading).

Ctar-195 and Ctar-525 represent 2 canonical OBP protein sequences, transcripts of which were found expressed in the salivary glands of *C. tarsalis*. Homologs of these canonical OBP proteins were also previously found expressed in *C. quinquefasciatus *and *An. gambiae *sialotranscriptomes. These proteins tend to be much more conserved in sequence to mosquito and even *Drosophila *proteins and it is possible that they may play an endogenous or housekeeping function.

#### The 30 kDa antigen/GE rich/Aegyptin family

This protein family is typical of the salivary glands of adult female mosquitoes, and was first identified as a salivary antigen in *Ae. aegypti *[42], and later found in salivary transcriptomes and proteomes of both culicine and anopheline mosquitoes [4,5,35-37,43,44], where it was named GE rich protein. Distantly related proteins also exist in black flies and sand flies. More recently, proteins of this family from *Aedes *and *Anopheles *were shown to prevent platelet aggregation by collagen [14,45], indicating conservation of function after the split of the culicidae into the culicines and anophelines, approximately 150 MYA [46].

**Figure 3:**
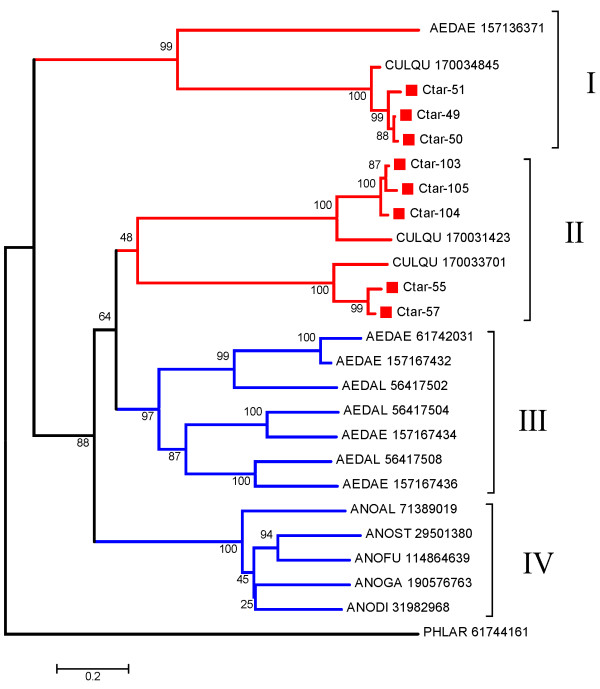
**Phylogram of the 30 kD/GE rich/Aegyptin protein family of mosquitoes**. The numbers on the tree nodes represent the percent bootstrap support in 10,000 trials. The bar at the bottom indicates 20% amino acid divergence. The *Culex tarsalis *sequences are named Ctar-XXX where the XXX represents the cluster number that originated it. The remaining sequences are named in a five letter followed by number format where the 3 first letters represent the 3 first letters of the genus, followed by the first 2 letters of the species binomial name. The number represents the NCBI gi| access. For more details, see text.

The sialotranscriptome of *C. tarsalis *allowed the identification of 8 protein sequences from the 30 kDa antigen/Aegyptin family (Additional file 2, Table S2), all represented by 1-10 ESTs found in the library. Blast searches against the NR database retrieved closely related sequences. Those that were more than 95% identical and of the same species were excluded to avoid inclusion of alleles or very closely related genes. Clustal alignment of this subset was used to produce a bootstrapped phylogram (Fig 3) that allows the following inferences: The 8 proteins from *C. tarsalis *most probably derive from 3 genes, Ctar-49, Ctar-50 and Ctar-51 being probable alleles of a gene closely related to a *C. quinquefasciatus *salivary gene shown in Clade I. Clade I includes *Aedes aegypti *salivary proteins that are short versions of the canonical 30 kDa protein, as are all *Culex *proteins in this clade. A second *C. tarsalis *gene possibly codes for Ctar-103, Ctar-104 and Ctar-105, and a third gene for Ctar-55 and Ctar-57. These two gene products groups with strong bootstrap support to their *C. quinquefasciatus *homologs, and these in turn group relatively weakly (48% bootstrap support) in Clade II as shown in Fig 3. Fig 3 also shows the uniquely *Aedes *containing proteins in clade III, represented by pairs of homologs between *Ae. aegypti *and *Ae. albopictus*, and the uniquely anopheline clade IV, with one sequence each of 5 different mosquito species. This phylogram suggests the single gene status of this family in the *Anopheles *genus, and the multi gene character in Culicines. At least 3 genes for canonical 30 kDa Ag/Aegyptin exists in the *Aedes *genus, and at least one more gene for the shorter protein shown in Clade I. *Culex *also have the shorter version gene, plus at least 2 genes of the canonical type.

#### Mucins and Peritrofins

Serine and threonine rich proteins are a common finding in sialotranscriptomes. These proteins are generally modified post-translationally and their mature forms have N-acetyl galactosamine residues, typical of mucins [47]. They probably have a function to lubricate the food canals and may also have antimicrobial function. Ctar-246 and Ctar-429 encode related protein sequences that might reflect splice variants of the same gene. These 2 truncated protein sequences have over 60 predicted glycosylation sites, and are similar to a previously described salivary mucin of *C. quinquefasciatus*. Ctar-261 is related to *Aedes *and *Culex *mucins that, in the case of the *Aedes *protein, were associated with induction by viral infection suggesting an immune function for this protein with 13 putative glycosylation sites. Ctar-581 encodes a 5' truncated protein sequence similar to salivary mucins of *C. quinquefasciatus *and *Aedes albopictus*.

#### Enzymes

Additional file 1, Table S1 indicate the presence of transcripts coding for enzymes possibly associated with the sugar meal and blood meal. Related to the sugar meal are several transcripts coding for glycosidases similar to proteins annotated as maltase and amylase. Additional file 2, Table S2 provides one full length sequence of a salivary alpha-glucosidase similar to other Culicine salivary maltases, plus 4 other truncated sequences coding for different sugar hydrolases. Regarding blood meal-related enzymes, 4 EST's coding for fragments of adenosine deaminase were found, as well as for an endonuclease. In *C. quinquefasciatus *a salivary endonuclease has been previously characterized and associated with helping forming the feeding hematoma [48]. However, the *C. tarsalis *enzyme is most closely related to sand flies salivary endonucleases and only distantly related to the salivary endonuclease of *C. quinquefasciatus*, although it is closely related to a non-salivary *C. quinquefasciatus *enzyme, indicating this *C. tarsalis *enzyme may be playing a housekeeping function.

Remarkably absent from the *C. tarsalis *sialotranscriptome are ESTs coding for members of the 5' nucleotidase, which functions as the salivary apyrase of mosquitoes. Apyrase hydrolyzes ATP and ADP to AMP and orthophosphate, destroying these important agonists of inflammation and platelet aggregation [2,49]. It has been previously noticed that *C. quinquefasciatus *has little salivary apyrase activity when compared to *Aedes *and *Anopheles *mosquitoes, and this observation was postulated to be a consequence of the lack of platelets in birds, the most common host of *Culex *[50], while *Aedes *and *Anopheles *are mostly mammalian feeders [51]. This may explain the absence of 5'-nucleotidase/apyrase coding transcripts in *C. tarsalis*, although an increased sequencing effort could produce 5'-nucleotidases that might be secreted. As an example of the abundance of this type of transcript in mosquito sialotranscriptomes, only one transcript out of 503 clones coded for apyrase in the *C. quinquefasciatus *sialotranscriptome [5], while 99 out of 4,066 were found in a similarly made library from *An. gambiae*, and 66 out of 4,232 ESTs were found in the *Ae. aegypti *sialotranscriptome [4]. From these observed frequencies, the expected number of EST's for *C. tarsalis *and *C. quinquefasciatus *are 27 and 8, respectively, producing a Chi square of 54.6 and a P < 0.001, indicating the low expression of apyrase transcripts in *Culex*.

Three ESTs coding for a phosphatase produce the predicted truncated sequence encoded by Ctar-194, which is similar to a salivary alkaline phosphatase previously described in *Ae. aegypti*. The function of this enzyme in feeding, if any, is unknown.

Transcripts coding for at least 6 different serine proteases were found in *C. tarsalis *sialotranscriptome (Additional files 1 and 2, Tables S1 and S2). These enzymes may function in immunity as prophenoloxidase activators, or in digesting skin matrix components, such as in an elastase function, or hydrolysing host blood clotting enzymes such as fibrinogen/fibrin, or activating plasminogen.

#### Immunity related products

Antimicrobial peptides, lysozyme, and pathogen pattern recognition polypeptides are commonly found in the sialotranscriptome of blood sucking arthropods. Additional file 2, Table S2 shows the full length sequence of *C. tarsalis *salivary lysozyme, which is 91% identical to the *C. quinquefasciatus *homolog, and 75% identical to the salivary homolog of *Ae. albopictus*. Truncated ORF's of a C-type lectin and a Gram-negative binding protein were also found. They both match previously described salivary proteins of *Aedes *and *Culex*.

#### Secreted proteins with unknown function

##### Promiscuous Antigen 5 (AG-5) family

This is a ubiquitous protein family found in animals and plants [52], and in all sialotranscriptomes of blood sucking Diptera analyzed so far. The function of these proteins in mosquito saliva is not known, but in blood sucking Brachycera two proteins of this family have been functionally characterized. Remarkably, in a tabanid fly, a member of the AG-5 family acquired a typical RGD domain surrounded by Cys residues and acts as a main platelet aggregation [53], and in the stable fly a salivary AG-5 protein binds immunoglobulins and may function as an inhibitor of the classical complement pathway [54]. We present evidence, in the form of truncated transcripts, for the expression of at least two members of the family in *C. tarsalis *salivary glands; Ctar-151, assembled from 3 ESTs, matches with 94% identity the salivary secreted antigen-5 precursor AG5-3 from *Culex quinquefasciatus *while Ctar-438 matches with 91% identity another *C. quinquefasciatus *protein of the same family.

##### Insect specific Cys-Rich polypeptide family

Two transcripts were found in the *C. tarsalis *sialotranscriptome coding for basic (pI = 8.8) peptides of mature MW of 9.7 kDa containing 12 Cys residues. This peptide family was previously found in the salivary transcriptome of *C. quinquefasciatus*, but close relatives of the same size exist in *Drosophila*, *Bombyx*, *Tribolium *and *Apis*. The ubiquity of this protein family in insects, its size and pI suggests an antimicrobial role.

##### Hematophagous Diptera specific 41.0 kDa family

The first 41.0 kDa family member was characterized in the sialotranscriptome of *Ae. aegypti*, and later found in *C. quinquefasciatus *and in *Ae. albopictus *[4,5,36,55]. Although not present in the sialotranscriptomes of members of the anopheline Cellia subgenus *An. funestus, An. stephensi *and *An. gambiae *(including scaning of the deducted proteome), it was recently found in *An. darlingi*, a member of the Nyssorhynchus subgenus [56], characterizing this family as uniquely Culicidae. Additional file 2, Table S2 provides evidence of a member of this protein family in *C. tarsalis*, encoded by Ctar-541, producing a predicted protein of mature MW of 43 kDa, being 69% identical to its *C. quinquefasciatus *homolog. Psiblast of Ctar-541 against the NR protein database retrieves on its first blastp cycle only mosquito salivary proteins, as expected. On the second cycle, it retrieves with lower significance (e value > 0.005) mostly bacterial proteins, but also salivary proteins from *Simulium vittatum *and *Culicoides sonorensis*. Further iterations of Psiblast retrieves with high significance bacterial proteins of the methyl-accepting chemotaxis receptor family (MCP), which may suggest this bacterial family to be originated from horizontal gene transfer to an ancestral blood feeding Nematocera. The gene structure of this protein in *C. quinquefasciatus *and *Ae. aegypti *is similar, containing 2 exons with a short intron of ca. 60 nt. Inclusion of Simulium and Culicoides sequences in this family indicates an ancient origin before the Nematocera split [57].

##### Mosquito specific 23.4 kda protein

Ctar-345 codes for a protein related to mosquito proteins so far only found expressed in mosquito adult salivary glands. Psiblast of Ctar-345 against the NR database does not retrieve any additional protein with an e value better than 0.02.

##### Mosquito specific hyp37 family

This member of this mosquito salivary protein family was identified in *An. stephensi *[35], and later found also *An. gambiae *[3], but not previously in other mosquito transcriptomes, including *C. quinquefasciatus *and *Ae. aegypti*. Ctar-769, represented by a single EST in our database, codes for a member of this family as indicated by blastp comparisons to the NR protein database, where it retrieves only Culex and Anopheles proteins. Psiblast of Ctar-769 against the NR database does not increase finding matches beyond additional mosquito proteins, including proteins deducted from the uncovering of the *C. quinquefasciatus *genome with and without signal peptide, and additional proteins from *An. gambiae *lacking a signal peptide indicative of secretion. Interestingly, the *Anopheles gambiae *gene found expressed in the salivary glands was shown to reside as a single exon in chromosome arm 2R [3]. The *An. gambiae *gene was also found to be selectively expressed in the adult female salivary glands suggesting a role in blood feeding [3].

##### Culicine specific 30.5 kDa family

**Figure 4:**
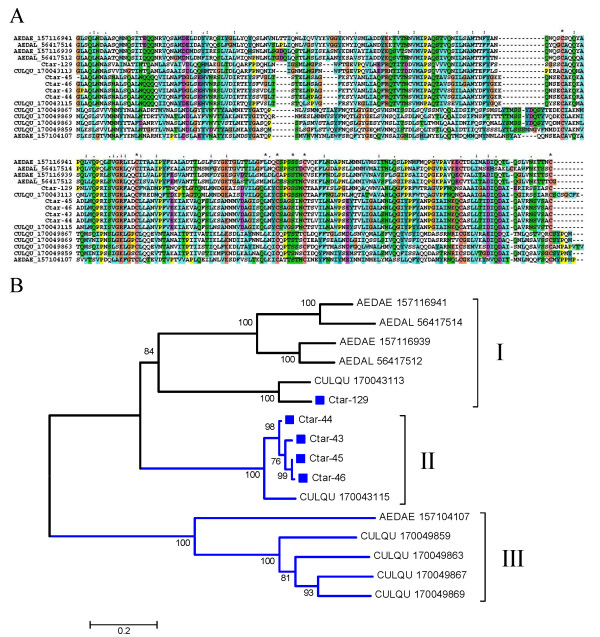
**The 30.5 kDa family of culicine proteins**. A) Clustal alignment. The *Culex tarsalis *proteins are identified by their Ctar-prefix. The remaining sequences are named with the first three letters from the genus name followed by two letters from the species name and by their NCBI protein accession number. The symbols above the alignment indicate: (*) identical sites; (:) conserved sites; less conserved sites. B) Neighbor Joining bootstrapped phylogram of the alignment in A. The *Culex tarsalis *proteins are marked with a square symbol. The numbers on the branches represent the percent bootstrap support. The bar in the bottom represents 20% amino acid divergence. For more details, see text.

Members of the 30.5 kDa protein were previously discovered in *Ae. aegypti, Ae. albopictus *and *C. quinquefasciatus *sialotranscriptomes. Additional related proteins were also deducted from the genomes of *Ae. aegypti *and *C. quinquefasciatus*, but not *An. gambiae*. The sialotranscriptome of *C. tarsalis *provides additional evidence for this multi copy family exclusive of Culicines. Clustal alignment of these protein sequences (Fig 4A) shows many conserved amino acids and a conserved Cys framework on the second half of the protein. The bootstrapped phylogram shows 3 robust clades. Clade I contains a sub clade of 2 *Ae. aegypti *and 2 *Ae. albopictus *proteins, and a second sub clade of a *C. quinquefasciatus *and Ctar-129. Clade II has 4 *C. tarsalis *sequences, possibly alleles of a single gene or closely related genes, and one *C. quinquefasciatus *sequence. Clade III shows a possible gene expansion in *C. quinquefasciatus *containing 4 gene products, clustering with one *Ae. aegypti *sequence. Overall the phylogram indicates that *Aedes aegypti *has at least 3 genes coding for this protein family, while *C. quinquefasciatus *has at least 6, 3 of which possibly arrived by further gene duplications within clade III. *Culex tarsalis *and *Ae. albopictus*, have at least two genes expressing these proteins in their adult female salivary glands. Psiblast of members of this protein family against the NR protein database converges after 6 iterations, retrieving solely *Aedes *and *Culex *proteins (not shown). The function of this protein family is unknown, but transcripts for this family were found enriched in the salivary glands of adult female *Ae. aegypti *[4] suggesting a blood feeding role.

##### Culicine specific 7.8 kDa family

**Figure 5:**
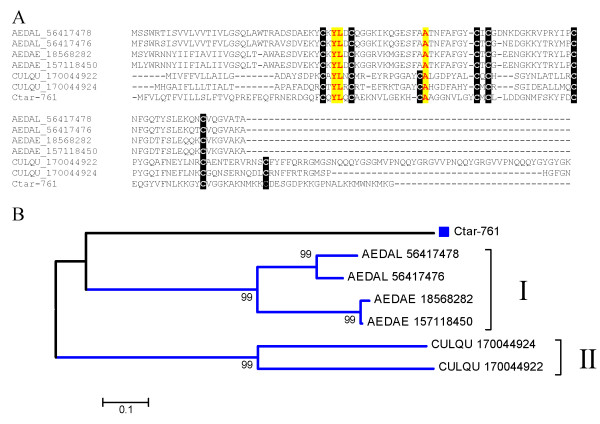
**The 7.8 kDa family of culicine peptides**. A) Clustal alignment. The *Culex tarsalis *proteins are identified by the square symbol and their Ctar-prefix. The remaining sequences are named with the first three letters from the genus name followed by two letters from the species name and by their NCBI protein accession number. Notice conserved cysteine framework in black background and identical amino acids in yellow or gray background. B) Neighbor Joining bootstrapped phylogram of the alignment in A. The numbers on the branches represent the percent bootstrap support. The bar in the bottom represents 10% amino acid divergence. For more details, see text.

Ctar-761 codes for a mature peptide of predicted MW = 11.7 kDa and pI = 8.8. It is similar to previously described salivary peptides found in sialotranscriptomes of *Ae. aegypti *and *Ae. albopictus*, but not *Culex*. Psiblast using an inclusion e value of 0.1 (relatively high to compensate for the small size of the sequences) against the NR protein database additionally identifies a salivary peptide previously found in *C. quinquefasciatus*, annotated as 9.7 kDa salivary peptide. Inclusion of this peptide with the previous to generate the reverse position search model [32] additionally identifies other salivary peptides from *C. quinquefasciatus*, with convergence of the search and identification of the 6 non redundant peptides with an e value of 8 e^-16 ^or smaller. Alignment of these 6 peptides with Ctar-761 shows that the *Aedes *sequences are more compact than those from Culex, containing a conserved framework of 6 cysteines, while those of *Culex *have 2 additional Cys residues (Fig 5a). Only 3 other residues are identical in all sequences, indicating a high evolutionary rate of this peptide family, if they indeed share a common ancestor. The bootstrapped phylogram (Fig 5B) shows 2 robust clades, clade I containing only *Aedes *sequences in two subclades, each containing a pair of *Ae. aegypti *or *Ae. albopictus *sequences. Clade II has 2 sequences from *C. quinquefasciatus*. Ctar-761 does not cluster with either of the clades, indicating again the possible fast evolutionary rate of this peptide family.

##### Culicine specific 4.2 kDa peptide family

Ctar-146, Ctar-147 and Ctar-208 encode peptides with similarities to *C. quinquefasciatus *4.2 kDa salivary peptide, which has similarity solely to another salivary peptide previously found in *Ae. albopictus*.

##### Culex genus specific cysteine and tryptophan rich protein family (CWRP)

**Figure 6:**
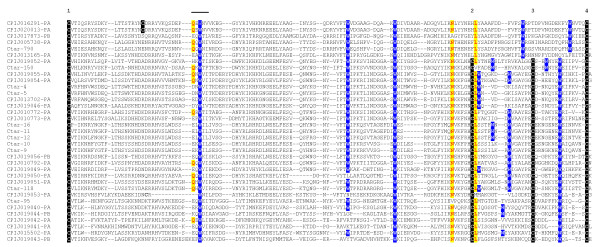
**The cysteine tryptophane rich proteins (CWRP) of Culex**. The number above the alignment indicates the conserved cysteine framework. The bar indicates the conserved Q-X-W motif found in lectin families. Also shown are the conserved tryptophane, asparagine and leucine residues. The *C. tarsalis *proteins are indicated by the prefix Ctar-. The *C. quinquefasciatus *proteins were obtained from the Vectorbase proteome prediction.

This protein family was previously discovered in the *C. quinquefasciatus *sialotranscriptome [5], where 10 proteins were deduced from the EST sequences. These have a signal peptide indicative of secretion, 4 conserved cysteins, 3 conserved tryptophans and one conserved asparagine and leucine residues. Inspection of the *C. quinquefasciatus *genome indicates a total of 30 genes coding for this protein family, most (28) members being single exonic. Expression of these genes in *C. quinquefasciatus *salivary glands was confirmed by Edman degradation of electrophoretically separated salivary homogenate [5]. The *C. tarsalis *sialotranscriptome led to identification of 6 full length members of this protein family, plus 14 other fragments. Alignment of the members of this family (Fig 6) shows a conserved cysteine framework in most members, two conserved tryptophan residues (in addition to 4 other less conserved tryptophan residues), as well at the conserved asparagine and leucine residues. This protein family is by far the most transcribed in *C. tarsalis *adult female salivary glands, accounting for 25% of the transcripts that were sequenced. Interestingly, the most transcribed member in *C. tarsalis*, Ctar-1, is a truncated member of the family by relatively recent acquisition of a stop codon just after the second conserve Cysteine. This abundant expression suggests that this family may be responsible for host serotonin or histamine antagonism, as is performed by the abundantly expressed D7 proteins of *Aedes *or *Culex*. This possibility is further supported by the relatively low expression of the D7 protein family in *C. tarsalis *and their absent serotonin binding motifs which are found both in *Aedes *and *Anopheles *D7 proteins (see above description of the D7 proteins). Psiblast of members of the CWRP protein family retrieves, interestingly, sugar binding proteins including bacterial glycosidases, lectins such as ricin and hemolysins, the crystal structure of which have a trefoil structure [58]. The single exon structure of this protein family in *C. quinquefasciatus *suggests either acquition of this gene family by horizontal transfer, and/or gene duplication occurring by retroposition of an RNA template.

##### Culex genus specific 9.7 peptide family

Ctar-520 and Ctar-29 encode truncated peptides matching the previously described putative 9.7 kDa salivary peptide of *C. quinquefasciatus*, which has no other matches to known proteins.

##### Culex genus specific GQP repeat protein family

Ctar-31, Ctar-32 and Ctar-33 produce matches to a low complexity protein previously described in the *C. quinquefasciatus *sialome characterized by a poly histidine repeat in the mature aminoterminal region, and a series of GQP/GQG repeats. The polyhistidine domain may confer a bacteriostatic role for this protein if it chelates Zn ions, a bacterial growth factor, and a domain existing in various antimicrobial peptides [59-61].

### Comparison of protein sequence identities between *C. tarsalis *and *C. quinquefasciatus *gene products

Over 170 deduced protein sequences coding for putative housekeeping (H) products are presented in Additional file 2, Table S2. These proteins allow comparison of the evolutionary rate of the S proteins compared to the H proteins, using the *C. quinquefasciatus *proteome as a reference set, as done before for comparing *An. stephensi *salivary proteins to those of *An. gambiae *[35]. For this comparison, we used only protein sequences from *C. tarsalis *that had at least 100 AA of alignment by the blastp tool to a *C. quinquefasciatus *protein, and excluded from this set possible alleles or closely related gene duplications by removing the smaller sequence(s) that had 80% or more similarity to another one within the set. We thus compared 50 putative secreted *C. tarsalis *proteins with their *C. quinquefasciatus *proteome, obtaining an average of 70.1% protein identity, while 169 putative housekeeping proteins from *C. tarsalis *were 91.2% identical to *C. quinquefasciatus *predicted proteins (see Additional file 2, Table S2 worksheets - P < 0.001 Mann-Whitney rank sum test). This significant difference further supports the concept that the evolution of mosquito salivary secreted proteins occur at a faster pace than housekeeping proteins, as indicated before for anopheline proteins [35,37,56].

It has been suggested that codon volatility (the proportion of the point-mutation neighbors of a codon that encode different amino acids) could be a measure of selection for fast evolution of proteins, as could occur in pathogens in a constant avoidance of antibody recognition [62]. Although this intuitive idea has created strong opposition [63-65], it is supported by published models [66,67]. We accordingly decided to measure the average codon volatility for the 205 sequences coding for housekeeping and 80 sequences coding for putative secreted proteins shown in Additional file 2, Table S2. The average codon volatility for the H class genes was 0.7609 + 0.001 while the S class had an average volatility of 0.7746 + 0.002 (Average + SE), a highly significant result (P = 6.9 × 10^-8^, double tailed t test). Whatever the discussion regarding the value of this index, it indicates that a single point mutation on an S class gene has a significantly higher chance of producing a non-synonymous amino acid substitution than in an H class gene.

## Conclusions

From a conservative perspective, the sialotranscriptome of *C. tarsalis *confirms the presence of ubiquitous salivary mosquito protein families, such as the D7, 41 kDa, hyp37, 30 kDa antigen/Aegyptin, 23.4 kDa, mucins, cysteine rich peptide, antigen 5, amylase/maltase, and the immunity related proteins lysozyme, pathogen recognition molecules, and serine proteases possibly associated with immunity or with host matrix/fibrin degradation. Adenosine deaminase has also been found, but not the 5'nucleotidase/apyrase that has been abundantly found in mammalian feeding mosquitoes, but possibly absent or reduced in the bird feeding *C. tarsalis*. From another stand point, the *C. tarsalis *sialotranscriptome confirmed the presence of proteins so far known exclusively in culicine mosquitoes, such as endonucleases and the 62 kDa, 30.5 kDa, 7.8 kDa and 4.2 kDa families, all without a known function. Additionally, further confirmation of unique proteins of the Culex genus were found for members of the highly expanded CWRP, 9.7 kDa and the GPQ repeat families.

It has been previously indicated that many unique mosquito protein families appear to be related to bacterial proteins, (when using the Psiblast tool against the NR database) suggesting their acquisition by horizontal transfer. Interestingly, the genes coding for such proteins, now available for *An. gambiae*, *C. quinquefasciatus *and *Ae. aegypti *are often single exonic, as is the case of bacterial genes. In the case of *C. tarsalis*, this appears to be the case with the 41 kDa (which have a single short intron) and the CWRP (mostly uniexonic in *C. quinquefasciatus*) families. However, other possibilities for single exonic genes are their acquisition as gene duplication resulting from retrotransposition of an mRNA precursor deriving from an endogenous, multi exonic gene. At any rate, the frequency of the relatively unusual processes of horizontal transfer and/or retrotransposition in the acquisition of new genes associated with blood feeding appear to occur at a high rate in the formation of mosquito sialomes.

Since our transcriptome was based on ~2,000 ESTs from a non-normalized library, the question arises as to what extent novel putative secreted proteins can be discovered with a more extensive sequencing, or the use of a normalized library. As indicated in a recent review [68], it has been our experience that, perhaps due to the relatively low complexity of the salivary gland proteome (when compared to organs like the mammalian liver or brain, for example), 1000-2000 sequenced clones are enough to display the majority of the sialome. Indeed, ~2,000 clones sequenced from an *Ae. aegypti *salivary gland cDNA library [4] discovered virtually all those found in another effort that obtained ~20,000 sequences [69]. A similar situation was encountered with the *An. gambiae *sialome, where ~4,000 sequences identified basically all those in a large sequencing effort, also ~20,000 sequences done by the Pasteur Institute [3]. Sequencing of normalized libraries, or more intensive sequencing of existing libraries, perhaps with newer generation of pyrosequencing methodology, may increase the number of salivary secreted peptides, but possibly to no more than 10% of the number found with random sequencing of ~2,000 clones. It should be indicated, however, that these additional low abundance transcripts may account for pharmacologically potent peptides.

Finally, the fast divergence of salivary proteins allows the possibility of using *C. tarsalis *proteins as specific markers of vector exposure, as is being attempted for ticks [70-72], mosquitoes [26-29] and sand flies [73].

## Methods

### Mosquitoes and cDNA library construction

*Culex tarsalis*, strain Bakersfield (Bakersfield, California) was supplied by Dr. W.K. Riesen University of California-Davis. Colonized mosquitoes were maintained on mouse blood (for egg laying) and given 10% sucrose ad libitum. Larvae were reared and adults maintained under controlled conditions of temperature (27°C), humidity (70% RH), and light (16:8 L:D diurnal cycle). PolyA+ RNA was extracted from 80 dissected pairs of salivary glands using the Micro-FastTrack mRNA isolation kit (Invitrogen, Carlsbad, CA), which was then used to make a non-normalized PCR-based cDNA library using the SMART™ cDNA library construction kit (BD Biosciences-Clontech, Palo Alto, CA) as described before [55].

### cDNA sequencing

The salivary gland cDNA library was plated on LB/MgSO4 plates containing X gal/IPTG to an average of 250 plaques per 150 mm Petri plate. Recombinant (white) plaques were randomly selected and transferred to 96 well MICROTEST TM U bottom plates (BD BioSciences, Franklin Lakes, NJ), containing 100 μl of SM buffer (0.1 M NaCl; 0.01 M MgSO4; 7 H2 O; 0.035 M Tris HCl (pH 7.5); 0.01% gelatin) per well. The plates were covered and placed on a gyrating shaker for 30 min at room temperature. The phage suspension was either immediately used for PCR or stored at 4°C for future use.

To amplify the cDNA using a PCR reaction, 4 μl of the phage sample was used as a template. The primers were sequences from the λ TriplEx2 vector and named pTEx2 5seq (5/TCC GAG ATC TGG ACG AGC 3/) and pTEx2 3LD (5/ATA CGA CTC ACT ATA GGG CGA ATT GGC 3/), positioned at the 5/end and the 3/end of the cDNA insert, respectively. The reaction was carried out in 96 well flexible PCR plates (Fisher Scientific, Pittsburgh, PA) using TaKaRa EX Taq polymerase (TAKARA Mirus Bio, Madison, WI) on a GeneAmp^® ^PCR system 9700 (Perkin Elmer Corp., Foster City, CA). The PCR conditions were: one hold of 95°C for 3 min; 25 cycles of 95°C for 1 min, 61°C for 30 sec; 72°C for 5 min. The amplified products were analysed on a 1.5% agarose/EtBr gel. cDNA library clones were PCR amplified, and the ones showing single band were selected for sequencing. Approximately 200-250 ng of each PCR product was transferred to Thermo Fast 96 well PCR plates (ABgene Corp., Epsom, Surray, UK) and frozen at -20°C before cycle sequencing. Samples were shipped on dry ice to the Rocky Mountain Laboratories Genomics Unit with primer and template combined together in an ABI 96-well Optical Reaction Plate (P/N 4306737) following the manufacturers recommended concentrations. Sequencing reactions were setup as recommended by Applied Biosystems BigDye^® ^Terminator v3.1 Cycle Sequencing Kit by adding 1 μl ABI BigDye^® ^Terminator Ready Reaction Mix v3.1 (P/N 4336921), 3 μl 5× ABI Sequencing Buffer (P/N 4336699), and 2 μl of water for a final volume of 10 μl. Cycle sequencing was performed at 96°C for 10 seconds, 50°C for 5 seconds, 60°C for 4 minutes for 27 cycles on either a Bio-Rad Tetrad 2 (Bio-Rad Laboratories, Hercules, CA) or ABI 9700 (Applied Biosystems, Inc., Foster City, CA) thermal cycler. Fluorescently-labeled extension products were purified following Applied Biosystems BigDye^® ^XTerminator™ Purification protocol and subsequently processed on an ABI 3730xL DNA Analyzer (Applied Biosystems, Inc., Foster City, CA). The AB1 file generated for each sample from the 3730xL DNA Analyzer was provided to researchers in Rockville, MD through a secure network drive for all subsequent downstream sequencing analysis. In addition to the sequencing of the cDNA clones, primer extension experiments were performed in selected clones to further extend sequence coverage.

### Bioinformatic Tools and Procedures

Expressed sequence tags (EST) were trimmed of primer and vector sequences. The BLAST tool [32], CAP3 assembler [74] and ClustalW [75] software were used to compare, assemble, and align sequences, respectively. For assembly of ESTs, a pipeline using blast and the CAP3 assembler was used, by first blasting all sequences using blastn with a word size of 36 and feeding the results as a fasta file to the CAP3 assembler[76]. The CAP3 parameters were set at default values, with an overlap length cut off of 40 and percent identity of overlap at 80%. Phylogenetic analysis and statistical neighbor-joining (NJ) bootstrap tests of the phylogenies were done with the Mega package [77]. For functional annotation of the transcripts we used the tool BlastX [32] to compare the nucleotide sequences to the non-redundant (NR) protein database of the National Center for Biotechnology Information (NCBI, National Library of Medicine, NIH,) and to the Gene Ontology (GO) database [33]. The tool, reverse position specific Blast (RPSBLAST) [32]was used to search for conserved protein domains in the Pfam [78], SMART [79], Kog [80], and conserved domains databases (CDD) [81]. We have also compared the transcripts with other subsets of mitochondrial and rRNA nucleotide sequences downloaded from NCBI and to several organism proteomes downloaded from NCBI, ENSEMBL, or VectorBase. Segments of the three-frame translations of the EST (because the libraries were unidirectional, 6-frame translations were not used), starting with a methionine found in the first 300 predicted amino acids (AA), or the predicted protein translation in the case of complete coding sequences, were submitted to the SignalP server [82] to help identify translation products that could be secreted. O-glycosylation sites on the proteins were predicted with the program NetOGlyc [83]. Functional annotation of the transcripts was based on all the comparisons above. Following inspection of all these results, transcripts were classified as either Secretory (S), Housekeeping (H) or of Unknown (U) function, with further subdivisions based on function and/or protein families. Codon volatility was calculated as previously described [62].

## Abbreviations

aa: amino acid; AMP: antimicrobial peptide; AG5: antigen 5 family; EST: expressed sequence tag; H class: housekeeping; kbase: kilobase; NR: nonredundant; nt: nucleotide; OBP: odorant binding protein; S class: secreted; SG: salivary gland; SMART: switching mechanism at 5/end of RNA transcript; U class: unknown function.

## Authors' contributions

EC helped with library manufacture, sequencing, data analysis, and contributed to the manuscript. AJF and KDB. helped with sequencing the cDNA. VMP participated in sequencing the library. JMCR performed data analysis, and contributed to the manuscript. IS-V and KEO provided biological specimens and contributed to the manuscript. All authors read and approved the final manuscript.

## Supplementary Material

Additional file 1 **Table S1**. Excel file with EST assembly results. Click here for file

Additional file 2 **Table S2**. Excel file with deducted protein sequences. Click here for file
